# Supplementary material for: BplMYB46 from Betula platyphylla Can Form Homodimers and Heterodimers and Is Involved in Salt and Osmotic Stresses
Source: Int J Mol Sci. 2019 Mar 7;20(5):1171. doi: 10.3390/ijms20051171 (PMC6429157; doi:10.3390/ijms20051171)
Supplement: Supplementary file 1 [file ijms-20-01171-s001.zip › supplementary files/╕╜═╝.docx]

Figure S1. Multiple sequence alignment of BplMYB46 and other MYBs obtained using ClustalW.


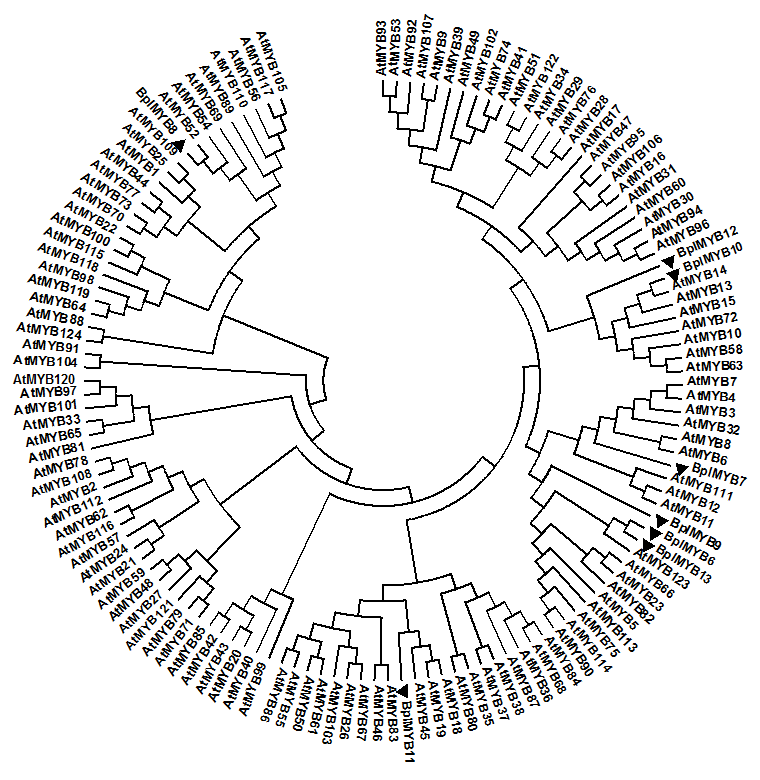


Figure S2. Phylogenetic analysis of MYB protein sequences. The full-length amino acid sequences of eight *B. platyphylla* and all *Arabidopsis* MYB were aligned by Clustalx 1.83, and the un-rooted Neighbor-Joining tree was constructed using MEGA 5.1 with 1000 bootstrap replicates. The sequences of the Arabidopsis MYB domain proteins were downloaded from the *Arabidopsis* genome TAIR 9.0 (http://www.arabidopsis.org/).
