# Supplementary material for: BplMYB46 from Betula platyphylla Can Form Homodimers and Heterodimers and Is Involved in Salt and Osmotic Stresses
Source: Int J Mol Sci. 2019 Mar 7;20(5):1171. doi: 10.3390/ijms20051171 (PMC6429157; doi:10.3390/ijms20051171)
Supplement: Supplementary file 1 [file ijms-20-01171-s001.zip › supplementary files/╕╜▒φ.docx]

**Table S1 Sequence characteristics of eight MYB proteins**

| Name | The length of amino acids | Molecular weight of proteins (kDa) | Isoelectronic  point |
| --- | --- | --- | --- |
| BplMYB6 | 284 | 32.3 | 6.80 |
| BplMYB7 | 240 | 27.2 | 8.15 |
| BplMYB8 | 244 | 28.9 | 8.51 |
| BplMYB9 | 300 | 33.5 | 8.66 |
| BplMYB10 | 270 | 30.6 | 5.38 |
| BplMYB11 | 290 | 32.8 | 6.35 |
| BplMYB12 | 341 | 38.8 | 5.49 |
| BplMYB13 | 262 | 29.7 | 8.17 |

**Table S2 Primer sequences used to construct prey and bait expression vectors**

| Primer | Sequence (5’-3’) |
| --- | --- |
| BD-MYB1-1 | CATGGAGGCCGAATTCATGGGGAGGAGTCCATGCTG |
| BD-MYB1-2 | GCAGGTCGACGGATCCCGGCCACTCCTGATAATCAAGG |
| BD-MYB2-1 | CATGGAGGCCGAATTCATGAGGAAACCTTGCTGTGAG |
| BD-MYB2-2 | GCAGGTCGACGGATCCATATTGATCTTTTCCTGTTT |
| BD-MYB3-1 | CATGGAGGCCGAATTCATGTGCACCAGAGGCCACTG |
| BD-MYB3-2 | GCAGGTCGACGGATCCAGAACTTCCATTAACAGAT |
| BD-MYB4-1 | CATGGAGGCCGAATTCATGGGAAGGGCTCCTTGTTG |
| BD-MYB4-2 | GCAGGTCGACGGATCCTCAGATCAATAGCGACTCTGC |
| BD-MYB5-1 | CATGGAGGCCGAATTCATGGTGAGAGCTCCATGCTG |
| BD-MYB5-2 | GCAGGTCGACGGATCCAAAATCTGGTAATTCTGGT |
| BD-MYB6-1 | CATGGAGGCCGAATTCATGGGGTTCAAGTCATCAGAC |
| BD-MYB6-2 | GCAGGTCGACGGATCCGTACATTACATTATTATTCAT |
| BD-MYB7-1 | CATGGAGGCCGAATTCATGGTGAGAACTCCTAGCTG |
| BD-MYB7-2 | GCAGGTCGACGGATCCATACATAGATTTCTCCATGC |
| BD-MYB8-1 | CATGGAGGCCGAATTCATGGGTAGAAGCCCTTGCTG |
| BD-MYB8-2 | GCAGGTCGACGGATCCTTATTCAACCTGCCATTCT |
| AD-MYB1-1 | CAACGCAGAGTGGCCATTATGGCCCATGGGGAGGAGTCCATGCTG |
| AD-MYB1-2 | TCTAGAGGCCGAGGCGGCCGACATGCGGCCACTCCTGATAATCAAGG |
| AD-MYB2-1 | CAACGCAGAGTGGCCATTATGGCCCATGAGGAAACCTTGCTGTGAG |
| AD-MYB2-2 | TCTAGAGGCCGAGGCGGCCGACATGATATTGATCTTTTCCTGTTT |
| AD-MYB3-1 | CAACGCAGAGTGGCCATTATGGCCCATGTGCACCAGAGGCCACTG |
| AD-MYB3-2 | TCTAGAGGCCGAGGCGGCCGACATGAGAACTTCCATTAACAGAT |
| AD-MYB4-1 | CAACGCAGAGTGGCCATTATGGCCCATGGGAAGGGCTCCTTGTTG |
| AD-MYB4-2 | TCTAGAGGCCGAGGCGGCCGACATGTCAGATCAATAGCGACTCTGC |
| AD-MYB51 | CAACGCAGAGTGGCCATTATGGCCCATGGTGAGAGCTCCATGCTG |
| AD-MYB5-2 | TCTAGAGGCCGAGGCGGCCGACATGAAAATCTGGTAATTCTGGT |
| AD-MYB6-1 | CAACGCAGAGTGGCCATTATGGCCCATGGGGTTCAAGTCATCAGAC |
| AD-MYB6-2 | TCTAGAGGCCGAGGCGGCCGACATGGTACATTACATTATTATTCAT |
| AD-MYB7-1 | CAACGCAGAGTGGCCATTATGGCCCATGGTGAGAACTCCTAGCTG |
| AD-MYB7-2 | TCTAGAGGCCGAGGCGGCCGACATGATACATAGATTTCTCCATGC |
| AD-MYB8-1 | CAACGCAGAGTGGCCATTATGGCCCATGGGTAGAAGCCCTTGCTG |
| AD-MYB8-2 | TCTAGAGGCCGAGGCGGCCGACATGTTATTCAACCTGCCATTCT |
| AD-MYB46-F | CAACGCAGAGTGGCCATTATGGCCCATGAGGAAGCCGGAGGCCT |
| AD-MYB46-R | TCTAGAGGCCGAGGCGGCCGACATGCTGAACTTGGAAATCAAGT |

**Table S3 Primer sequences used to construct plant overexpression pROK2-BplMYBs vectors**

| Primer | Sequence (5’-3’) |
| --- | --- |
| p1301-MYBCORE-F | AGCTTCAGTTACAGTTACAGTTAACCCTTCCTCTATATAAGGAAGTTCATTTCATTTGGAGAGAACACGGC |
| p1301-MYBCORE-R | CATGGCCGTGTTCTCTCCAAATGAAATGAACTTCCTTATATAGAGGAAGGGTTAACTGTAACTGTAACTGA |
| MYB6-F | CTCTAGAGGATCCCCATGGGGAGGAGTCCATGCTG |
| MYB6-R | TCGAGCTCGGTACCCTCACGGCCACTCCTGATAAT |
| MYB8-F | CTCTAGAGGATCCCCATGTGCACCAGAGGCCACTG |
| MYB8-R | TCGAGCTCGGTACCCTTAAGAACTTCCATTAACAG |
| MYB11-F | CTCTAGAGGATCCCCATGGGGTTCAAGTCATCAG |
| MYB11-R | TCGAGCTCGGTACCCTTAGTACATTACATTATTAT |
| MYB12-F | CTCTAGAGGATCCCCATGGTGAGAACTCCTAGCTG |
| MYB12-R | TCGAGCTCGGTACCCTTAATACATAGACTTCTCC |
| MYB13-F | CTCTAGAGGATCCCCATGGGTAGAAGCCCTTGCTG |
| MYB13-R | TCGAGCTCGGTACCCTTATTCAACCTGCCATTCTC |

**Table S4 Primer sequences used in the chromatin immunoprecipitation-PCR (ChIP-PCR)** **analysis**

| Primer | Sequence (5’-3’) |
| --- | --- |
| MYB6-121F | CTAGCCCGGGATGGGGAGGAGTCCATGCTG |
| MYB6-121R | GTACACTAGTCGGCCACTCCTGATAATCAAG |
| MYB8-121F | GCTCTAGAGCATGTGCACCAGAGGCCACTG |
| MYB8-121R | GTACACTAGTAGAACTTCCATTAACAGATA |
| MYB11-121F | GCTCTAGAGCATGGGGTTCAAGTCATCAG |
| MYB11-121R | GTACACTAGTGTACATTACATTATTATTCAT |
| MYB12-121F | GCTCTAGAGCATGGTGAGAACTCCTAGCTG |
| MYB12-121R | GTACACTAGTATACATAGACTTCTCCATGC |
| MYB13-121F | GCTCTAGAGCATGGGTAGAAGCCCTTGCTG |
| MYB13-121R | GTACACTAGTTTCAACCTGCCATTCTCCT |
| MYB46-121F | GCTCTAGAGCATGAGGAAGCCGGAGGCCTC |
| MYB46-121R | GTACACTAGTCTGAACTTGGAAATCAAGC |
| MYBCORE-F | GATTACGATTTTTTAATTTG |
| MYBCORE-R | TAACAGTGCAAATGTTGGCT |
| M46p-F | GATAATGTGAAGTGGAAGC |
| M46p-R | AGGTTGTCGTCTCGTCGTG |

**Table S5** **Primer sequences of six MYBs and internal control genes analyzed using real-time reverse transcription (RT)-PCR**

| Primer | Sequence (5’-3’) |
| --- | --- |
| *MYB46-F* | TCAGGTGGAGGTGAGAAA |
| *MYB46-R* | CAAGAAGGGAGTGCAAAT |
| *MYB6-F* | TGATCCCAGCAGTACCAT |
| *MYB6-R* | CAGAGTCTCCAGCACAAG |
| *MYB8-F* | CGTCAAAGATTTGCCTAC |
| *MYB8-R* | TCTATCACTTCGCTACCAT |
| *MYB11-F* | GCTAACCGCAGCTTCTTA |
| *MYB11-R* | GTCGCTATACTTTCCATCATTC |
| *MYB12-F* | CTCCTCCATCTCCAAAGA |
| *MYB12-R* | TCCATAAATTGCTGCTCT |
| *MYB13-F* | GGAGGGCTTGAATAGAGG |
| *MYB13-R* | TTCGTCCGGTGAGATGTT |
| Ubiquitin F | GGAGGACAAGGTGGAGGG |
| Ubiquitin R | GATTGAGGGGAGGGATGC |
| α-tubulin F | TGGCTCGAATGCACTGTTGG |
| α-tubulin R | TCAACCGCCTTGTCTCTCAGG |

**Table S6 Primer sequences used in the analysis of target genes using real-time reverse transcription (RT)-PCR**

| Primer | Sequence (5’-3’) |
| --- | --- |
| SOD4-F | CGGAGGTCATATCAACCACTC |
| SOD4-R | CAGACCAAGCCACACCCAT |
| SOD6-F | CTTTGCTCTTTCCTCACTCT |
| SOD6-R | ACAACCCTCGCATTTCAT |
| POD9-F | AGGAACGAGAACGCTACTGC |
| POD9-R | AACACGATCCGATCTGATGC |
| POD10-F | GATGGCAGGGTATCTTTG |
| POD10-R | GCTTGGTTTATGGTGGG |
| GST-F | ATGAGTACGGCAAGAAAGGAG |
| GST-R | TTCAGCCTCAGCCACTACAACT |
